# Metabolite profiling and transcriptomic analyses demonstrate the effects of biocontrol agents on alkaloid accumulation in *Fritillaria thunbergii*

**DOI:** 10.1186/s12870-023-04459-6

**Published:** 2023-09-18

**Authors:** Xuemei Cheng, Dishuai Li, Zheng Jiang, Cheng Qu, Hui Yan, Qinan Wu

**Affiliations:** 1 College of Pharmacy, Nanjing University of Chinese Medicine, Nanjing, China; 2 Jiangsu Collaborative Innovation Center of Chinese Medicinal Resources Industrialization, Nanjing, China; 3 National and Local Collaborative Engineering Center of Chinese Medicinal Resources Industrialization and Formulae Innovative Medicine, Nanjing, China

**Keywords:** *Fritillaria thunbergii*, Biocontrol agents, Metabolic, Transcriptomic, Steroidal alkaloid biosynthesis

## Abstract

**Background:**

During *Fritillaria thunbergii* planting, pests and diseases usually invade the plant, resulting in reduced yield and quality. Previous studies have demonstrated that using biocontrol agents can effectively control grubs and affect the steroid alkaloids content in *F. thunbergii*. However, the molecular regulatory mechanisms underlying the differences in the accumulation of steroid alkaloids in response to biocontrol agents remain unclear.

**Results:**

Combined transcriptomic and metabolic analyses were performed by treating the bulbs of *F. thunbergii* treated with biocontrol agents during planting. Otherwise, 48 alkaloids including 32 steroid alkaloids, 6 indole alkaloids, 2 scopolamine-type alkaloids, 1 isoquinoline alkaloid, 1 furoquinoline alkaloid, and 6 other alkaloids were identified. The content of steroidal alkaloids particularly peimine, peiminine, and veratramine, increased significantly in the group treated with the biocontrol agents. Transcriptome sequencing identified 929 differential genes using biocontrol agents, including 589 upregulated and 340 downregulated genes. Putative biosynthesis networks of steroid alkaloids have been established and combined with differentially expressed structural unigenes, such as acetyl-CoA C-acetyl-transferase, acelyl-CoAC-acetyltransferase3-hydroxy-3-methylglutaryl-coenzyme A synthase, 1-deoxy-D-xylulose-5-phosphate reductor-isomerase, 2-C-methyl-D-erythritol-4-phosphate cytidylyltransferase and 4-hydroxy-3-methylbut-2-enyl diphosphate reductase. In addition, biological processes such as amino acid accumulation and oxidative phosphorylation were predicted to be related to the synthesis of steroid alkaloids. Cytochrome P450 enzymes also play crucial roles in the steroid alkaloid synthesis. The transcription factor families MYB and bHLH were significantly upregulated after using biocontrol agents.

**Conclusions:**

Biocontrol agents increased the steroid alkaloids accumulation of steroid alkaloids by affecting key enzymes in the steroid alkaloid synthesis pathway, biological processes of oxidative phosphorylation and amino acid synthesis, cytochrome P450 enzymes, and transcription factors. This study revealed the mechanism underlying the difference in steroidal alkaloids in *F. thunbergii* after using biocontrol agents, laying the groundwork for future industrial production of steroid alkaloids and ecological planting of medicinal materials in the future.

**Supplementary Information:**

The online version contains supplementary material available at 10.1186/s12870-023-04459-6.

## Background

*Fritillaria thunbergii*, a common herb of the Liliaceae family, has been used extensively in traditional medicine for up to 2000 years history [1]. *F. thunbergii* is widely distributed in China, including in the Zhejiang, Jiangsu, and Hunan provinces. As a medicine and food homologous to Chinese medicine, the bulb of *F. thunbergii* is rich in alkaloids, amino acids, nucleosides, and sugars that have multiple biological activities, including anti-tussive, anti-asthmatic effect and analgesic effect [2]. Therefore, it has an extremely high practical value and social benefit. The application and development of *F. thunbergii* have broad market prospects in the future.

Recently, the demand for *F. thunbergii* has increased markedly leading to a growing planting scale [3]. The incidence of pests and diseases is increasing annually. Grubs and root rot are the mainly pests and diseases, respectively, in the planting of *F. thunbergii*, and the current present prevention and control methods are primarily based on using chemical pesticides during the planting [4]. The use of chemical pesticides results in the degradation of soil, an imbalance in soil nutrients, changes in soil microbial flora, and a decline in soil enzyme activity [5]. Lately, biocontrol agents have attracted increasing attention and have been listed as environmentally friendly alternatives to synthetic agricultural chemicals. Biocontrol agents are not only beneficial microorganisms used to control plant diseases and pests, but also can improve the quality of plants [6]. Routinely used biocontrol agents include bacteria and fungi. As an endophytic entomopathogenic fungus, *Metarhizium anisopliae* has great potential for grub prevention. ‘Ning Dun’ is a biological bacterium, the main component of which is *Bacillus subtilis*, which prevents root rot and promotes production. Some endophytic fungi have been reported to induce the synthesis of host polysaccharides and alkaloids [7]. *Metarhizium anisopliae* and *B.* subtilis can promote the defense function and growth metabolism in host [8, 9]. According to earlier research, these two types of biocontrol agents (*Metarhizium* anisopliae and ‘Ning dun’) can affect the production of alkaloids in *F. thunbergii* [10]. However, the stress response of *F. thunbergii* following the application of biocontrol agents and the mechanisms affecting alkaloid synthesis remain unclear. The elucidation of this mechanism will be crucial for the cultivation of *F. thunbergii*.

The transcriptome is now routinely used to understand the molecular mechanism of functional representation of metabolite biosynthesis [11–13]. Additionally, the expression patterns of genes related to steroid alkaloid biosynthesis in Fritillaria plants can be determined through transcriptome sequencing [14–16]. Based on previous research and transcriptome sequencing on *F. thunbergii* to explore the internal influencing mechanism of alkaloid accumulation after treatment with the biocontrol agent.

Ultra-high-performance liquid chromatography-tandem mass spectrometry (UHPLC-MS/MS) was applied in the current investigation to identify the metabolites of *F. thunbergii* following the application of biocontrol agents. Changes in the expression of important genes connected to steroid alkaloids were discovered using transcriptome analysis. We also discussed whether and how biosynthesis is associated with amino acid synthesis and oxidative phosphorylation. This investigation lays the groundwork for a thorough comprehension of the bioactive component synthesis and regulatory mechanisms in *F. thunbergii.* The study is helpful for advancing the further advancement of the biological control of diseases and pests of *F. thunbergii*, which may provide a reference value for the ecological planting of medicinal materials in the future.

## Results

### UHPLC-MS/MS analysis of bioactive constituents in *F. thunbergii*

**Fig. 1:**
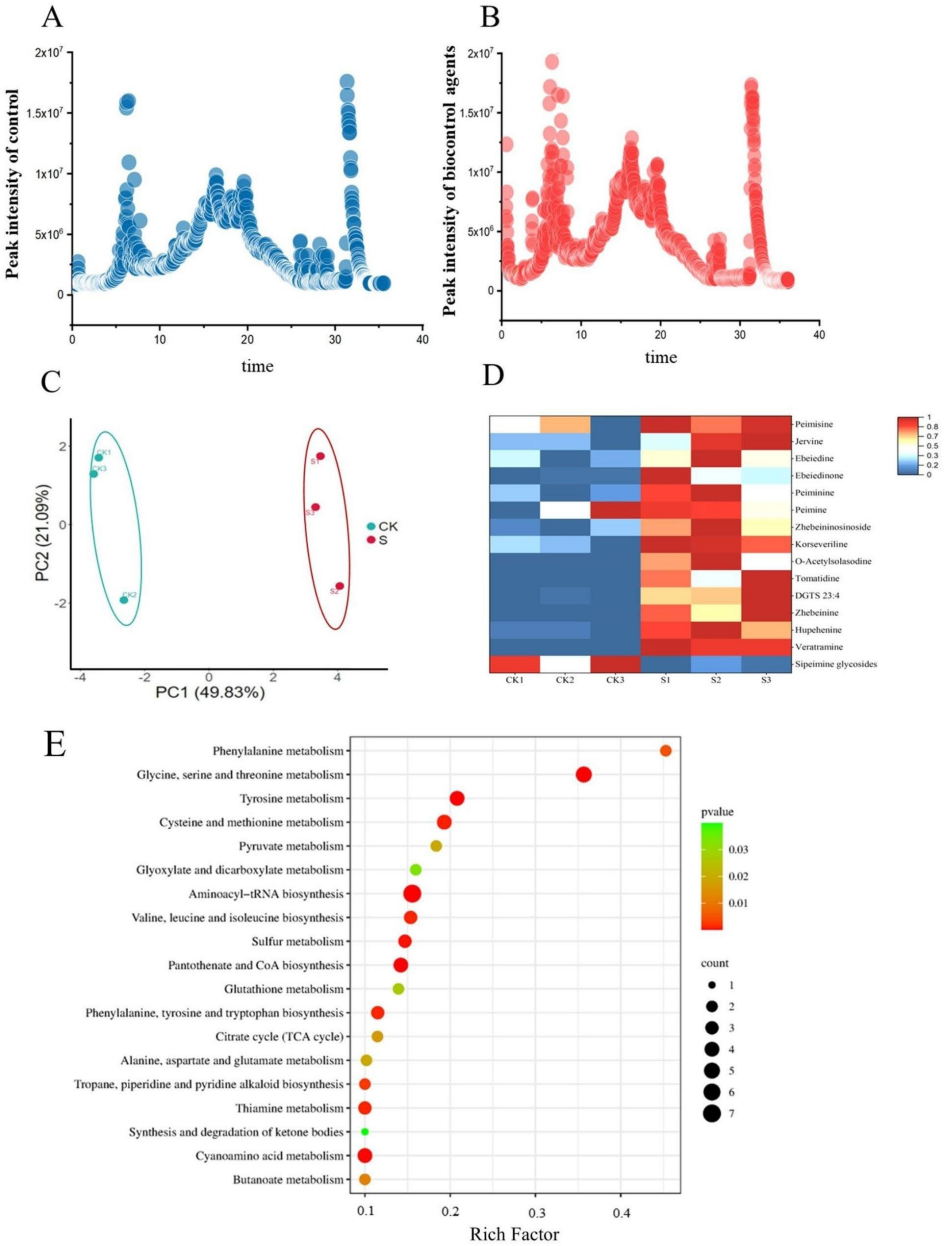
Distribution of bioactive constituents identified using ultra-high-performance liquid chromatography (UHPLC)-triple time-of-flight tandem mass spectrometry in **A** control group and **B** biocontrol agents group based on their retention time. **C** PCA of metabolomic data. **D** Heatmap of identified differential alkaloid metabolites (*p* < 0.05, VIP ≥ 1). **E** Bubble map of metabolite pathway enrichment analysis in the CK and S groups

The growth status and physiological indices of *F. thunbergii* treated with biocontrol agents are shown in Fig. S1. The application of the biocontrol agents significantly enhanced the growth of *F. thunbergii*, and the number of flowers during the same period exceeded that in the blank control group (CK) (Fig. S2). In order to study the differences in the components of the bulbs of *F. thunbergii* in the treated and untreated groups, crude extracts were analyzed by UHPLC triple TOF-MS/MS. Based on the study of the in conjunction with standards and associated literature Reports, a total of 165 components were identified in accordance with their *t*Rs, correct masses, and MS/MS fragmentation properties (Table S1). The results revealed that the metabolic product spectra of various components in the bulb of *F. thunbergii* in group S were different from those of the CK group (Fig. 1A, B). PCA showed that the three replicates of each set were clustered together and that the two groups were visibly distinguishable (Fig. 1C). A total of 48 alkaloids were identified in the CK and S groups, including 32 steroid alkaloids, 6 indole alkaloids, 2 scopolamine-type alkaloids, 1 isoquinoline alkaloid, 1 furoquinoline alkaloid, and 6 other alkaloids (Table S2). A total of 15 important differentially expressed metabolites (DEMs) were screened using OPLS-DA analysis based on VIP ≥ 1 and *p* < 0.05. The heat map displayed that the remaining alkaloids content in the S group was greater than that in the CK group, except for sipeimine glycosides (Fig. 1D). To better obtain the functions of metabolites with obviously differences between CK and S group, the KEGG enrichment analysis was performed. The DEMs were considerably more enriched in the aminoacyl-tRNA biosynthesis and glycine, serine, and threonine metabolism pathways as compared with the CK group.

### Transcriptome assembly and functional annotation

As illustrated in the Table S3, the raw readings obtained for each library ranged from 68.9 million to 87.2 million, and the clean readings ranged from 68.5 million to 86.8 million. The Q30% of each library was > 93% and the average GC content of all libraries was approximately 49%. In addition, 88.60–92.92% of clean reads were obtained from each library. Overall, the transcriptome data can be used for the next analysis.

**Fig. 2:**
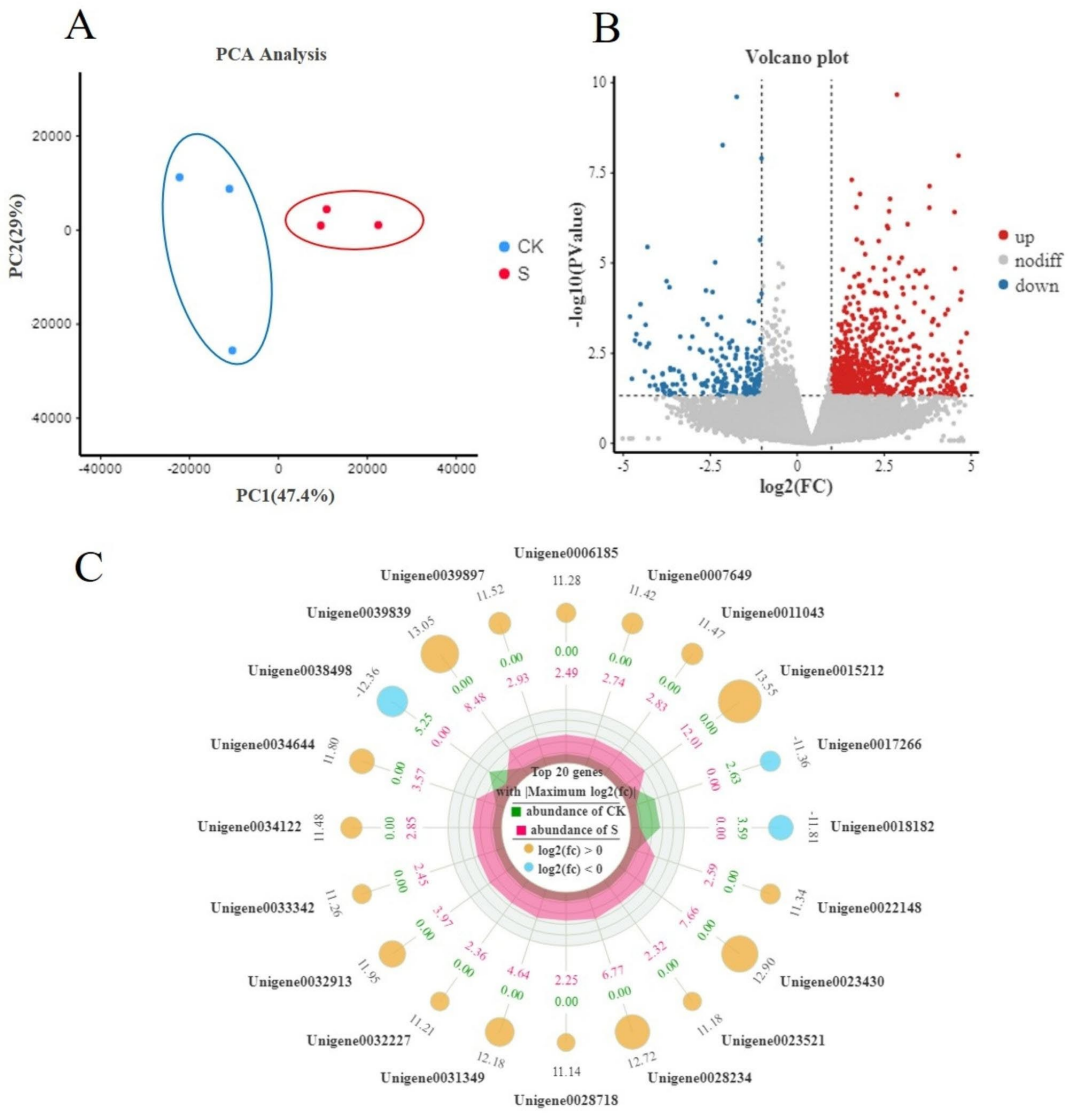
**A** Principal component analysis (PCA) of biocontrol agent and control groups. **B** Volcano map analysis of differentially expressed genes (Red, green, and gray points represent upregulated, downregulated, and unchanged genes, respectively). **C** Radar map of differential genes. (Yellow and blue represent upregulated, downregulated genes)

Principal component analysis (Fig. 2A and S3) revealed that the biological replicates of each set of samples were clustered in one region, and the two groups were significantly separated. This suggests that the two groups of samples differed significantly from one another. The higher the PC1 value, the greater was the difference before and after the application of biocontrol agents. The variance was divided into two main components, PC1 and PC2, which each accounted for 47.4% and 29% of the variance. Pairwise comparisons were performed with |log2 (fold change)| ≥ 1 and FDR < 0.05 as the threshold. In total, 929 differentially expressed genes (DEGs) were identified in the bacterial treatment group and control groups; of which, 589 were upregulated and 340 were downregulated (Fig. 2B). The radar map showed the information on the TOP 20 genes with the largest difference between the two groups (Fig. 2C). Green and red represent the average expression in the CK and S groups, respectively. As shown in the figure, the average expression of unigene0015212, unigene0039839 and unigene0023430 were higher in S group.

### Analysis of DEGs

**Fig. 3:**
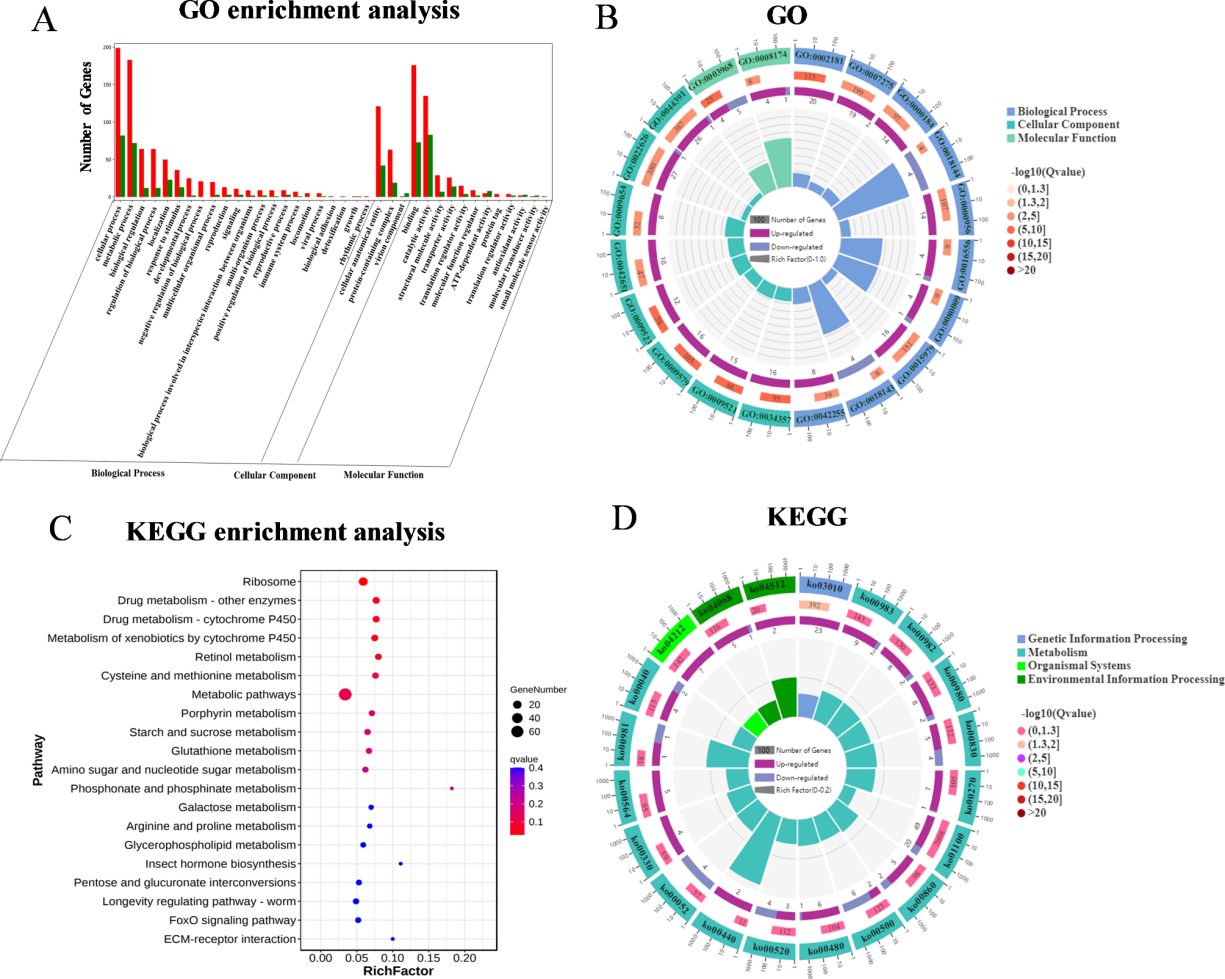
**A** Gene ontology (GO) enrichment of top 20 terms between CK and S groups. **B** Kyoto Encyclopedia of Genes and Genomes (KEGG) enrichment of top 20 pathways between CK and S groups. **C** The top 20 enrichment circle diagrams of GO. **D** The top 20 enrichment circle diagrams of KEGG.

By setting |log2 (fold change)| ≥ 2 and FDR < 0.05 as the thresholds for selecting significantly different genes, the RPKM value of each gene was calculated. The GO and KEGG functional annotation of all the DEGs were used for the two groups. The most enriched were cellular and metabolic processes (Fig. 3A), and 10 GO terms were enriched in the biological process section (Fig. 3B), (the coordinate scale for gene number is shown outside the first circle, and different colors stand for various ontologies). In the cell component module, the most enriched GO terms were cellular anatomical entity and proteins-containing complex (Fig. 3A), and eight GO terms were enriched in this part (Fig. 3B). The most enriched genes in the molecular function module were binding and catalytic activity-related genes with 2 GO terms. Based on the above results, following treatment with biocontrol agents, the metabolic processes and some enzyme activities in *F. thunbergii* changed; therefore, the alkaloid content changed [17]. Similarly, as shown in the Fig. 3 C and D, with an FDR value of < 0.05 considered as a significant enrichment, KEGG pathway annotations were mainly enriched in ribosomes (ko03010), drug metabolism-other enzymes (ko00983), drug metabolism-cytochrome P450 (ko00982), metabolism of xenobiotics by cytochrome P450 (ko00980), retinol metabolism (ko00830), cysteine and methionine metabolism (ko00270) and metabolic pathways (ko01100) [18–20]. The loop diagram shows that the ko01100 pathway was the most DEG with the most significant difference (Fig. 3D).

### Analysis of DEGs and metabolites related to steroid alkaloid synthesis pathway

**Fig. 4:**
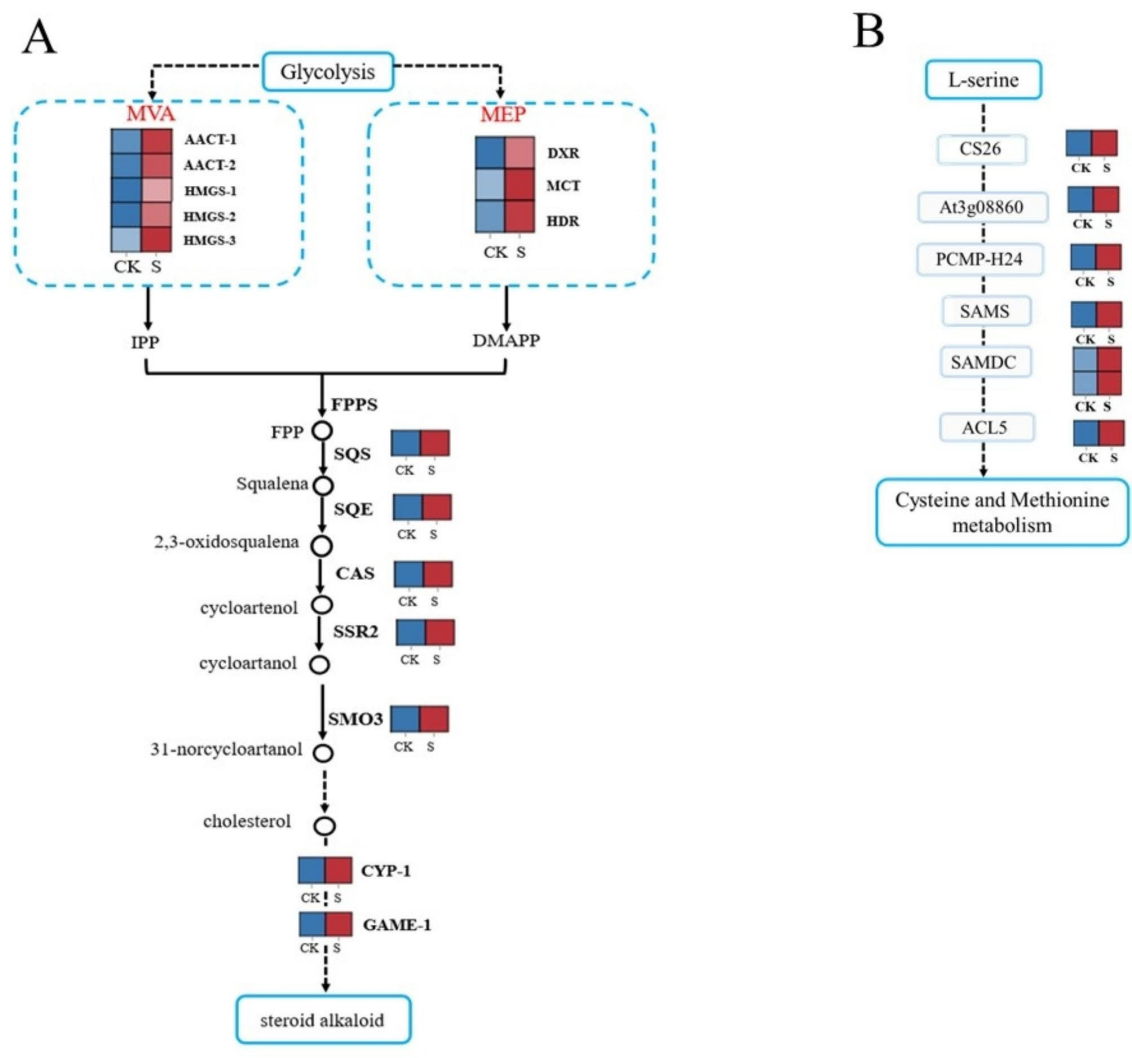
**A** Expression profiles of DEGs in the steroidal alkaloid biosynthesis pathway. **B** Differential expression levels of unigenes related to cysteine and methionine. Changes in expression level are represented by a change in color; Red color denoted genes with high expression level, while blue color denoted genes with low expression level. The relative expression levels of DEGs were calculated using Pearson’s correlation coefficient *p* < 0.05. All data shown reflect the average mean of three biological replicates

The putative enzyme genes and chemical structures of important intermediary chemicals involved in the production of steroid alkaloids in *F. thunbergii* were analyzed to propose the steroidal alkaloid synthesis pathway [18–20] (Fig. 4A, Table S4). The 15 genes were significantly upregulated in this pathway. AACT, HMGS, DXR, MCT, and HDR are key functional genes linked to the upstream synthesis pathway of steroid alkaloids. Beginning from acetyl coenzyme A, the genes encoding acetyl-CoA C-acetyl-transferase (AACT), acelyl-CoAC-acetyltransferase3-hydroxy-3-methylglutaryl-coenzyme A (HMG-CoA) synthase (HMGS), 1-deoxy-D-xylulose-5-phosphate reductor-isomerase (DXR), 2-C-methyl-D-erythritol-4-phosphate cytidylyl-transferase (MCT), 4-hydroxy-3-methylbut-2-enyl diphosphate reductase (HDR), which are involved in the 2-C- methyl-d-erythritol 4-phosphate (MEP) and Mevalonate (MVA) pathways, were highly enriched in the S group. One gene encoding cycloartenol synthase (CAS), a component of cycloartenol biosynthesis, was upregulated in the current study. Glycoalkaloid metabolism (GAME) was markedly increased in the downstream production pathway of steroid alkaloids; therefore, it is hypothesized that it was crucial in the synthesis of steroid alkaloids.

### DEGs involved in amino acid synthesis

In this study, a total of 7 DEGs involved in the amino acid synthesis pathway were identified (Fig. 4B, Table S5). Compared to the CK group, majority of the DEGs in the S group were upregulated, including Unigene0040075, Unigene0035737, Unigene0043166, Unigene0000588, Unigene0018323, Unigene0030949, and Unigene0010962. Among the top 20 KEGG pathways a total of 4 pathways were related to amino acids, which is consistent with our metabolome results [18–20]. Therefore, it can be concluded that the genes related to the synthesis of amino acids are closely related to the synthesis of steroidal alkaloids in *F. thunbergii*.

### DEGs involved in oxidative phosphorylation pathway

**Fig. 5:**
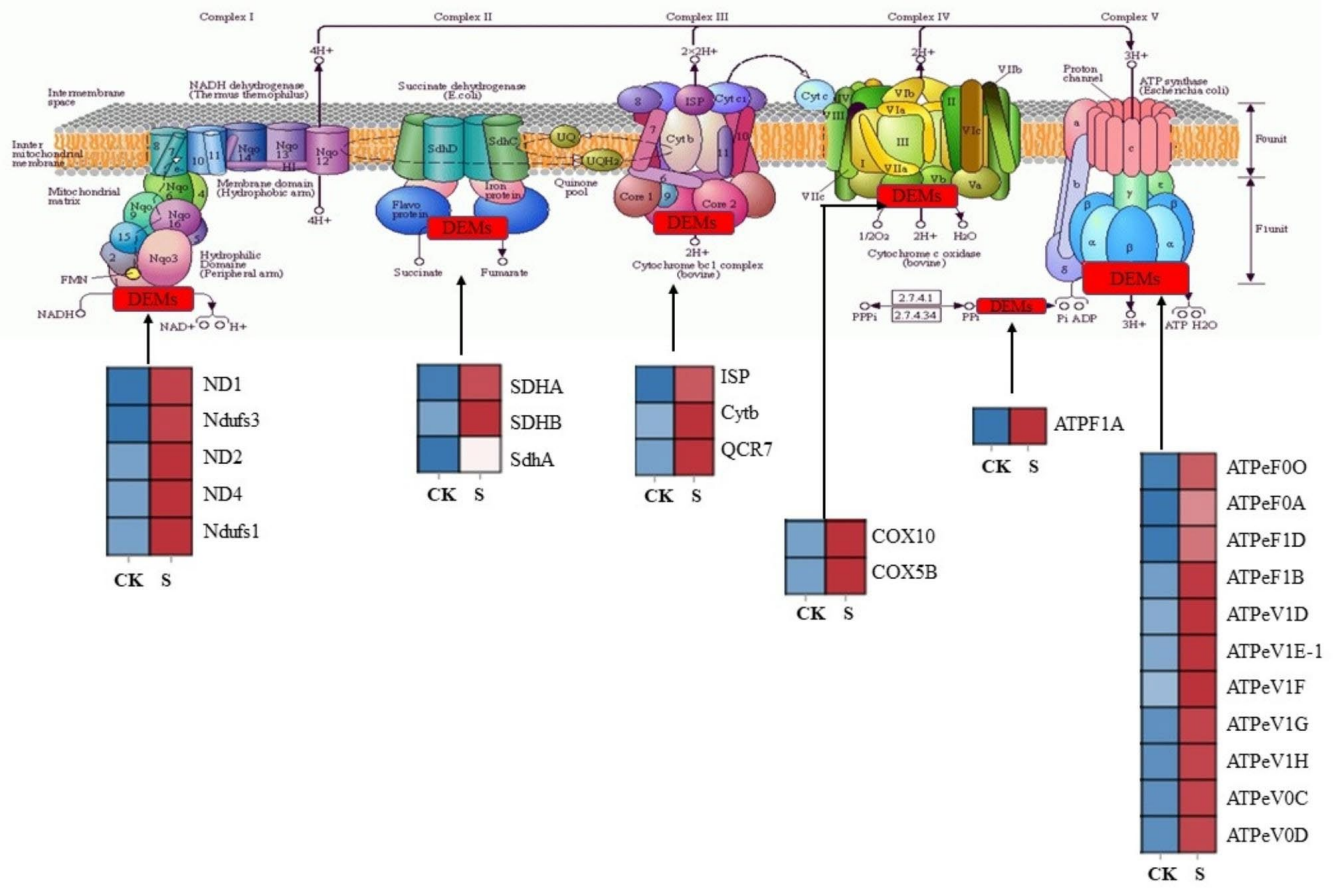
Expression profiles of DEGs in the oxidative phosphorylation pathway. Red color denoted genes with high expression level, while blue color denoted genes with low expression level. All data shown reflect the average mean of three biological replicates

In the present study, a total of 25 DEGs were identified by comparing CK and S groups. Most oxidative phosphorylation pathway genes were increased in the S group compared with the CK group (Fig. 5; Table S6). The gene expression of NADH dehydrogenase (ND1, Ndufs3, ND2, ND4, Ndufs1), ATP synthase (ATPeF0O, ATPeF0A, ATPeF1D, ATPeF1B, ATPeV1D, ATPeV1E-1, ATPeV1F, ATPeV1G, ATPeV1H, ATPeV0C and ATPeV0D), cytochrome c oxidase (COX10 and COX5B), soluble inorganic thermal phosphatase (ATPF1A) and cytochrome complex enzyme (ISP, Cytb and QCR7) were upregulated. The findings showed that the application of biocontrol agents may boost oxidative phosphorylation, raise the expression of these genes, and release more energy for the production of steroid alkaloids. This is similar to the results of a previous study [21].

### Differential expression of cytochrome P450s

In the present study, the 12 genes encoding cytochrome P450 enzymes (CYPs) were identified in the transcriptome of *F. thunbergii*. After applying the biocontrol agent, nine genes encoding CYPs were significantly upregulated, whereas three genes were significantly downregulated (Table S7), indicating that they had a positive regulatory effect on the expression of the steroid alkaloid synthesis pathway in *F. thunbergii* and played an important role in steroid alkaloids synthesis.

### Identification of DEGs in alkaloid synthesis using quantitative real-time PCR (qRT-PCR)

**Fig. 6:**
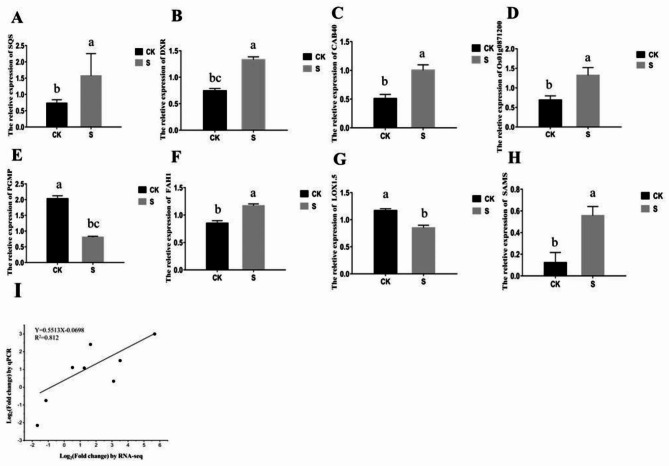
Difference in the expression levels of (**A**) SQS, (**B**) DXR, (**C**) CAB40, (**D**) Os01g0871200, (**E**) PGMP, (**F**) FAH1, (**G**) LOX1.5 and (**H**) SAMS between CK and S groups. (**I**) Correlation of the results between RNA-Seq and qRT-PCR.

The differential expression of 8 essential genes encoding important proteins of steroid alkaloids in untreated and biocontrol agent treated *F. thunbergii* samples was confirmed by qRT-PCR (Fig. 6, Table S8). The results revealed a high correlation co-efficient (R^2^ = 0.812) between the RNA sequencing data and qRT-PCR results, indicating that the RNA sequencing data were dependent (Fig. 6I).

### Transcription factor analysis

**Fig. 7:**
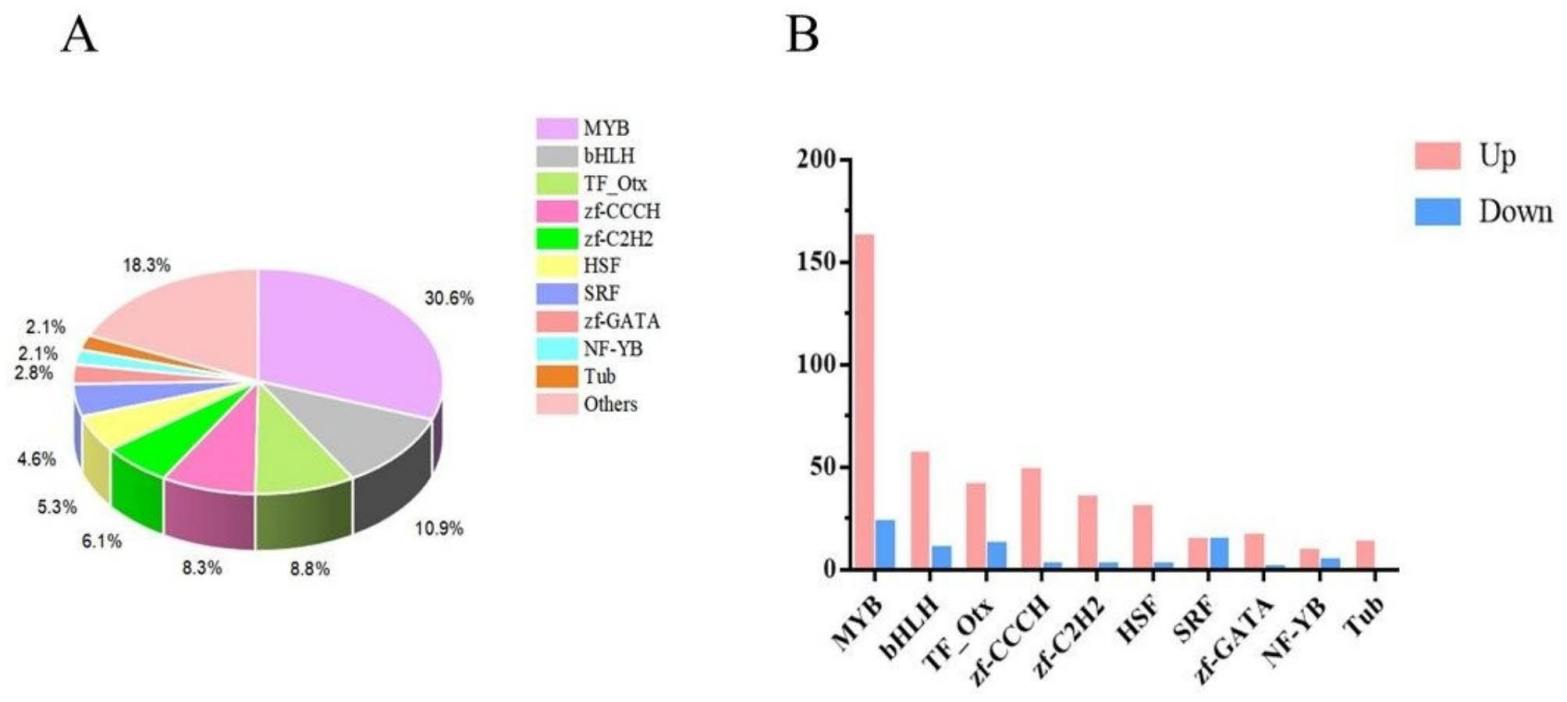
TFs DEGs involved in the synthesis of steroid alkaloids. **A** TFs. **B** Top10 TFs in samples under the CK and S systems

Transcription factors (TFs) control plant metabolism, germination, and responses to biotic and abiotic stressors [22, 23]. In our study, 605 differentially expressed TFs were annotated to the transcriptome of *F. thunbergii*, which was divided into 33 transcription factor families. The top 10 families were MYB (185), bHLH (66), TF-Otx (53), zf-CCCH (50), zf-C2H2 (37), HSF (32), SRF (28), zf-GATA (17), NF-YB (13), and Tubs (13) (Fig. 7). Notably, majority of the genes encoding MYB, bHLH, TF_Otx, zf-CCCH, zf-C2H2, and HSF were upregulated. These families are closely associated with the synthesis of carrier alkaloids. The MYB and bHLH transcription factor families are extensively distributed in plants and involve in a variety of functions, including floral organ development, plant hormone response, and fundamental metabolism and development of plants [24–27]. Therefore, the hormones and developmental metabolism of *F. thunbergii* undergo changes following the application of biocontrol agents.

## Discussion

At present, the research on the application of biological control agents during the planting process mainly focuses on the use of one biological control agent. Previous studies have reported that ‘Ning dun’ can control pests and diseases, and promote the growth during the cultivation of *F. thunbergii* [28]. *Bacillus subtilis* C3 can against bulb rot disease in *Fritillaria taipaiensis* P.Y.Li [4]. Endophytic fungi affected the accumulation of alkaloids by regulating the key enzyme genes in the alkaloid synthesis pathway of *Dendrobium officinale* [6]. Moreover, *Beauveria bassiana* can promote the growth of chive (*Allium schoenoprasum* L. [Amaryllidaceae]) and increase the alkaloid content of its active metabolites [29]. Overall, there are few studies on the combination of biocontrol agents during the planting process of *F. thunbergia*. In our previous studies, it was found that the combination of *Metarhizium anisopliae* and ‘Ning dun’ could not only control the pests and diseases during the cultivation of *F. thunbergii*, but also promote the accumulation of steroid alkaloids in *F. thunbergia* [10]. However, the molecular mechanism of its influence is unclear, which severely hinders the high-quality cultivation and effective utilization of *F. thunbergii*. In this study, we analyzed the internal molecular mechanism of *F. thunbergii* after using biocontrol agent using metabolomics and transcriptomics. Integrated transcriptomic and metabolomic analyses are effective tools for routinely evaluating the changes of active ingredients in medicinal plants [28]. The metabolomics results show that a total of 48 alkaloids were identified by using UHPLC-MS analysis, most of which were steroidal alkaloids. Peimine, peiminine, and peimisine contents in the microbial agent-treated group were higher than those in the CK group. Korseveriline, jervine, hupehenine, tomatidine, and veratramine levels in the microbial agent-treated group were higher than those of the CK group. Peimine, peiminine and peimisine are the most common active ingredients in *Fritillaria* plants [1]. Experimental evidence suggests that using biocontrol agents may stimulate the production of secondary metabolites, such as some amino acid-related metabolites and steroid alkaloids in *F. thunbergii*, thereby enhancing plant disease resistance. Alkaloids are secondary metabolites of plants to resist external stimuli and defend against pests and diseases [30].

Previous studies have suggested that the use of exogenous fungi promotes the accumulation of terpenoid alkaloids by affecting the MEP pathway [31, 32]. Combined with our transcriptome data, in comparison to the CK group, the rate-limiting enzyme DXR in the MEP pathway was markedly increased, indicating that more MEP accumulated with the application of biocontrol agents. MEP, as an important precursor in the pathway of steroid-alkaloid synthesis pathway, promotes the accumulation of steroid alkaloids. Simultaneously, the key enzymes MCT and HDR in the MEP pathway were also upregulated; therefore, more precursors of steroid alkaloid synthesis, IPP/DMAPP accumulated in the MEP pathway [33, 34]. Moreover, the synthesis of steroid alkaloids is also through the MVA pathway [32]. The key enzymes AACT and HMGS in the MVA pathway were markedly elevated in comparison to those in the CK group, indicating that more HMG-CoA and IPP intermediates may accumulate in *F. thunbergii* under the application of biocontrol agents [35]. A common precursor for the metabolism of phytosterols and cholesterol is provided by the cyclic artitenol synthase that is encoded by CAS [36]. Sterol side-chain reductase 2 (SSR2) converts cycloartenol to cycloartanol and is the first enzyme involved in the production of cholesterol. These key genes involved in terpenoid skeleton biosynthesis were upregulated, indicating that they may regulate the biosynthesis of alkaloids by affecting the supply of precursors. Additionally, in the downstream pathway of steroidal alkaloids’ synthesis, the expression of GAME-1 was markedly increased; therefore, it was speculated that GAME-1 plays a key role in the formation of steroidal alkaloids. It can be inferred from the results that most of the significantly different metabolites are strongly related to the biosynthesis of metabolic pathways and secondary metabolites.

Plant defense responses to biotic and abiotic stresses are largely dependent on amino acids [37]. From our transcriptome data, it is clear that the differential genes were abundant in the biosynthesis pathways of CYPs and amino acids. Among the top 20 KEGG enrichment pathways, the expression of genes related to arginine, cysteine, methionine and proline was upregulated. Previous studies have reported that arginine can enhance root development and improve the ability of crops to resist external stress [38, 39]. The addition of biocontrol agents stimulated the upregulation of genes related to amino acid synthesis in *F. thunbergii*, providing additional conditions for alkaloid synthesis It has previously been reported that cysteine maintains cell function and anti-oxidation and provides methyl groups for various compounds, including lipids, proteins, nucleic acids, alkaloids, and plant sterols [40]. Therefore, it is speculated that following the application of biocontrol agents, the cellular function of *F. thunbergii* became stronger, and more metabolites were synthesised. It has previously been reported that proline can improve the stress resistance of plants [41]. Our findings demonstrated that the quantity of proline in *F. thunbergii* increased, indicating that the stress tolerance in *F. thunbergii* was improved after the application of biological control agent, and the defense effect of foreign pests was enhanced. In addition, plants usually produce secondary metabolites, such as alkaloids and hormones, to enhance their immunity, thereby increasing the alkaloid content Studies have shown glutathione can help maintain normal immune system function and has an integrated detoxification function. This indicated that the addition of a biocontrol agent also affected the immune system function of fritillary plants. In conclusion, the upregulation of related genes and the synthesis of some amino acids in the steroid alkaloid synthesis pathway play important roles in the increase in steroid alkaloid content in biocontrol agents.

Besides, we observed that the application of biocontrol agents affected on the oxidative phosphorylation in *F. thunbergii* plants. Oxidative phosphorylation plays a key role in the decomposition and oxidation of organic matter in plant cells and can promote electron transfer and oxygen utilization utilisation to produce water and ATP [42]. Past investigations have discovered that the amassing of steroid alkaloids is not only related to their synthesis pathway, but also to the activation of the oxidative phosphorylation pathway [21]. In our study, the 25 related genes were markedly upregulated. Therefore, we speculate that the application of biocontrol agents promots the metabolism of oxidative phosphorylation and releases more energy to synthesise steroid alkaloids.

The largest enzyme family in plants is called CYPs [43]. They are essential to the higher plants’ production of antioxidants, phytohormones, and secondary metabolites. They also react to the crosstalk between abiotic and biotic stress responses. In this study, nine CYP-encoding genes were upregulated, and three genes were downregulated, indicating that CYPs may play a positive regulatory role in the biosynthesis of steroid alkaloids. In Fritillaria plants, steroid alkaloid production was regulated by MYB and bHLH [15, 44]. The majority of the genes encoding TF_Otx, zf-CCCH, zf-C2H2, and HSF-related, as well as genes associated to MYB, were elevated, suggesting that the high expression of these gene families may cause an accumulation of steroidal alkaloids in *F. thunbergii*. The relationships between CYPs, amino acids, TFs, and the formation of steroid alkaloids are complicated and need further investigation.

**Fig. 8:**
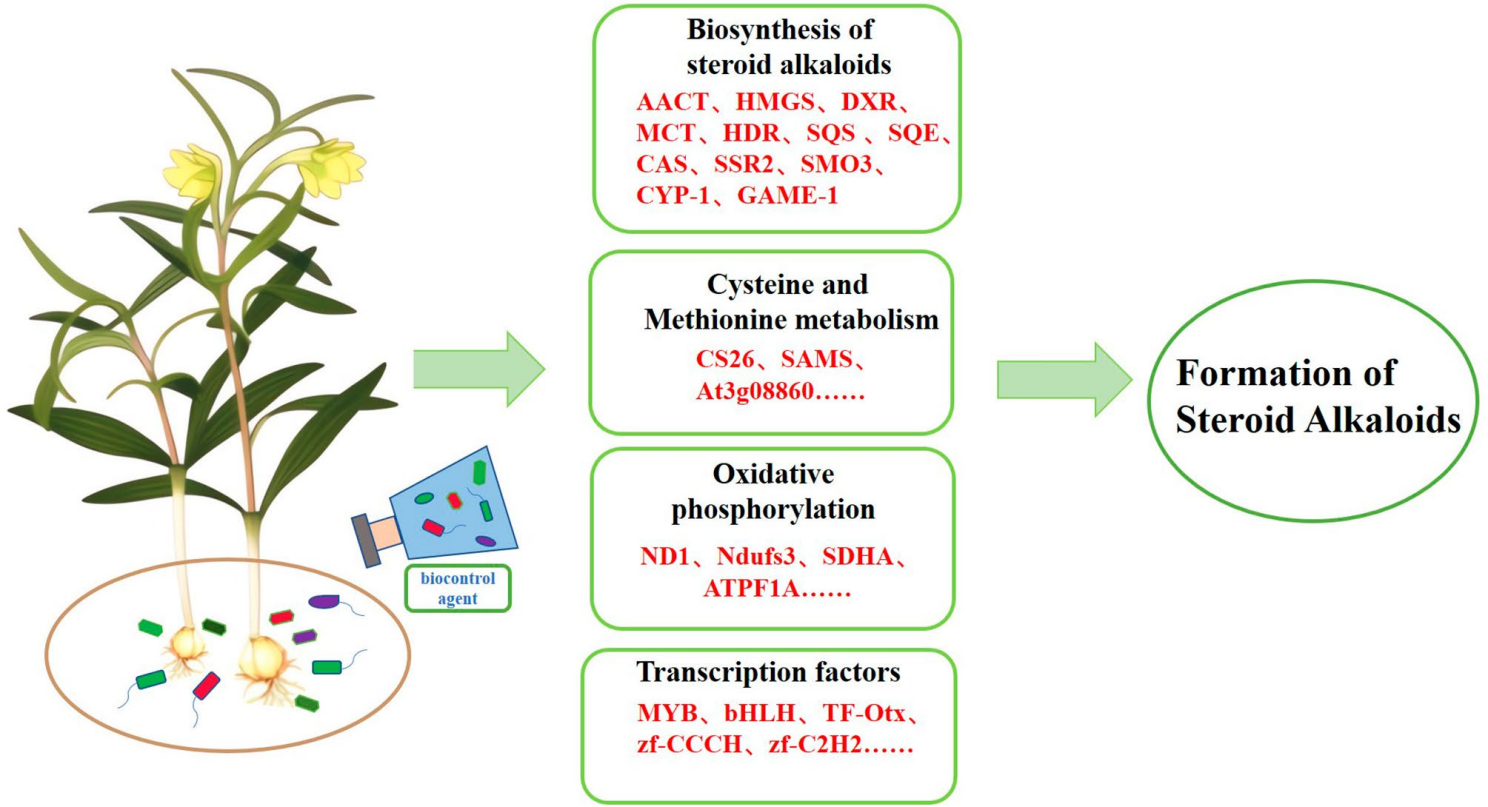
Proposed main molecular mechanisms of improving steroid alkaloids of *F. thunbergii* in response to the use of biocontrol agents

## Conclusions

In a word, this study revealed the potential mechanism underlying the increase in steroidal alkaloid content after the application of biocontrol agents. Fourteen differential metabolites in *F. thunbergii* were considerably upregulated under the influence of biocontrol agents. The differential genes of AACT, HMGS, DXR, MCT, and HDR were identified and showed a significant positive correlation with the synthesis of steroid alkaloids in *F. thunbergii* under the action of biocontrol agents. CYP-1 and GAME-1 were highly expressed; consequently, it was hypothesized that they might be involved in the steroid alkaloids’ downstream production routes. The synthesis of steroid alkaloids may also be impacted by biological processes like oxidative phosphorylation and amino acid buildup. Oxidative phosphorylation provides more energy and material basis for the synthesis of steroid alkaloids, and the accumulation of amino acids enhances the growth and development of *F. thunbergii* and its resistance to external stress. CYPs also play crucial roles in the synthesis of steroidal alkaloids. Transcription factor families, such as MYB and bHLH were significantly upregulated, promoting the metabolism of *F. thunbergii* and the accumulation of some endogenous hormones (Fig. 8). Currently, research on the complex symbiotic relationships between biocontrol agents and plants is insufficient, and there is great potential for the application and development of biocontrol agents in the future. This study provides a theoretical basis and ideas for the future use of biological control agents, development and substitution of chemical pesticides and provides a reference for the rational ecological planting of Chinese herbal medicine.

## Materials and methods

### Plant materials and biocontrol bacteria agent treatments

A field test of *F. thunbergii* planting base was conducted in the Libao Town, Nantong City, Jiangsu Province. (120°75′195′′E, 32°57′60′′N). The seeds (‘Zhebei 1’) of *F. thunbergii* were collected in Nantong, Jiangsu Province [10]. ‘Zhebei 1’ is a kind of breed selected by local growers over the years, which was identified as *F. thunbergii* by Professor Qinan Wu of Nanjing University of Chinese Medicine. Permission to collect *F. thunbergii* seeds was obtained from Nantong Zhulin Medical Technology Co., Ltd. The study protocol followed applicable guidelines and legislation at the institutional, national and international levels. The soil planted with *F. thunbergii* was loose, fertile, and near-neutral sandy loam. The land was then ploughed deeply into a turtleback ridge. The furrow was 150 cm wide and 20–25 cm deep. Each fritillary interval was approximately 20 cm for the buried soil planting. The literature has demonstrated the fundamental physical and chemical characteristics of the test soil. [10]. There was no additional effect on the alkaloid formation of *F. thunbergii*. In the experiment, *F. thunbergii* plants were treated with biocontrol agents after the seedlings were grown. The design of the two groups was based on previous studies. One group was treated with 0 kg/hm^2^ biocontrol agent (blank control group, CK), and the other group was treated with 150 kg/hm^2^ of the biocontrol agent (experimental group, S). Among the S group, *M.* anisopliae spore powder (CQMa128, Chongqing University, China) was 75 kg/hm^2^ + the ‘Ning dun’ (SM21, Nanjing Agricultural University, China) was 75 kg /hm^2^ [10]. The first dose was administered to S group on 5 March. The *M.* anisopliae spore powder was mixed with soil at a ratio of 1:10 into the field, and the ‘Ning dun’ was sprayed with water at a ratio of 1:500. The CK group was not treated with any biocontrol agents. Each group was set up three replicate groups and applied every half a month with three times. Finally, phosphate buffered saline (PBS) was used to rinse the mature *F. thunbergii* samples. One set was quickly frozen in liquid nitrogen after being cut into small pieces with disinfecting scissors for RNA sequencing. The other set was cut into sheets with disinfection knives and dried in an oven at 50 ℃ for metabolite determination.

### Metabolite profiling of bioactive constituents in *F. thunbergii*

All dried *F. thunbergii* samples were ground into a powder and each group was weighed precisely (1.0 g). Then it was extracted overnight with 10 mL of methanol. Then ultrasound for an hour. After shaking well and incubating for 5 min, 400 µL of the supernatant was collected, diluted with 400 µL of 75% acetonitrile, centrifuged at 12,000 rpm and 4 °C for 10 min, and then it was filtered through an organic membrane of 0.22 μm prior to undergoing the UHPLC-QTOF-MS/MS analysis [45].

The prepared solutions were analysed using UHPLC. A Waters XBridge™ C_18_ column (150 mm 2.1 mm, 2.5 m, USA) was used for the injection of aliquots (10 L) at a stable column temperature of 30 °C. With a flow rate of 0.4 mL/min, 0.1% aqueous formic acid (A) and acetonitrile (B) made up the UHPLC mobile phase. The following gradient elution parameters were used for chromatographic separation: 0 min, 2% B; 15 min, 45% B; 20 min, 80% B; 30 min, 100% B; and 36 min, 2% B. The injection volume was 5 L for both the samples and the reference substances.

High-resolution time-of-flight mass spectrometry Triple TOF™ 5600 (AB Sciex, Framingham, MA, USA), managed by Analyst TF 1.6 software (AB Sciex, Singapore), capable of electrospry ionization in both the positive and negative ion states. Each sample was scanned in the *m/z* 50–1200 Da range. The optimised MS parameters were as follows: ion source temperature was 400 ℃; ion spraying floating pressure was 4500v; ion source gas 1 was 60psi, ion source gas 2 was 60psi [46]. The collected data were analysed using MS-DIAL software (version 4.24) to identify potential chemicals in *F. thunbergii* samples from the control and microbial treatment groups. By establishing the analysis parameters in MS-DIAL, peak detection filtering, peak recognition, peak alignment smoothing, retention time correction, and metabolite labelling were automatically performed. In this study, the compounds were matched according to information in the MassBank of North America (mona.fiehnlab.ucdavis.edu/downloads) database and identified. The combined data (MSP format) were used as screening libraries. The average relative peak area of the compounds was quantified, and all analyses were performed three times [47]. These metabolites were annotated using public databases, including KEGG, HMDB, and LIPIDMaps database. Metabolites satisfying VIP ≥ 1 and *p* value < 0.05 were defined as DEMs between two groups.

### RNA isolation and cDNA Library Construction

According to the manufacturer ‘s instructions, total RNA was extracted from the frozen fritillary bulbs using the TRIzol reagent kit (Invitrogen, Carlsbad, USA) [48]. The enriched mRNA was fragmented into shorter fragments utilizing a fragmentation buffer, followed by reverse transcription into cDNA. Purified double-stranded cDNA fragments were end-repaired, added bases, and linked to Illumina sequencing adapters. And polymerase chain reaction (PCR) was then performed. Using an Illumina Novaseq6000 (Guangzhou, China), the resultant cDNA library was sequenced. To separate mRNA from total RNA for transcriptome information processing, A-T base-pairing oligo (dT) magnetic beads and ploy A (Invitrogen, Carlsbad, CA, USA) were utilized. Reverse transcription and random primers were used to create first-strand cDNA from the tiny fragments of purified mRNA, and then double-stranded cDNA was produced. Then, the end-repaired, ligated, and further fragmented double-stranded cDNA was purified [49]. Finally, the cDNA fragment was amplified using PCR, and a library of cDNA was constructed for Illumina sequencing [50].

### Transcriptome sequencing and functional annotation

First, low-quality sequences and contaminated adapters were filtered out of the raw data. Establishing a reference genome index and locating clean paired-end reads to the reference genome with HISAT2. [51]. Each sample’s mapped readings were put together using String Tie v1.3.1, a reference-based method [52]. RSEM software was used to calculate the fragment per kilobase of transcript per million mapped reads value in order to quantify the expression abundance and variations of each transcription region [53]. BLASTX was used to compare the non-redundant NCBI protein database (Nr, version 2019) [54], Swiss-Prot (version 2019). GO functional annotation was performed using Blast2go (version 2.5) and KOBAS (version 3.0), and related metabolic pathways were identified using KEGG.

### DEGs analysis

DESeq2 software was used to analyze the differential expression of RNAs between the two sets [54]. The |log2 (fold change)| ≥ 1 calculated using fragment per kilobase of transcript per million mapped reads value and FDR < 0.05 were used as the threshold for significant differences in unigene expression. The DEGs were enriched by GO analysis. In addition, DEGs engaged in significant KEGG pathways were found using KEGG enrichment analysis [55]. Based on the extensive KEGG pathway and gene functional annotation, we identified candidate DEGs encoding enzymes involved in steroid alkaloid production in order to investigate the various methods by which bioactive metabolites accumulate.

### qRT-PCR analysis

The relative mRNA expression patterns of these 8 genes (CAB40, PGMP, SQS, Os01g0871200, DXR, FAH1, SAMS and LOX1.5) were quantified using qRT-PCR, and the corresponding proteins were key enzymes involved in steroid alkaloid biosynthesis. The reaction system and qRT-PCR procedure were the same as in the previous study [56]. Table S9 contains a list of the qRT-PCR primers that were utilized.

### Statistical analysis

The TBtools software was used to generate heatmaps, while the Origin Pro 2021 software was employed for plotting graphs. SPSS version 24 was employed to perform paired-samples t-tests with a significance level of *p* < 0.05 to determine significant differences.

### Electronic supplementary material

Below is the link to the electronic supplementary material.

Supplementary Material 1

Supplementary Material 2

## Data Availability

The datasets generated during the current study are available in the NCBI database under SRA accession repository: PRJNA973832 (http://www.ncbi.nlm.nih.gov/bioproject/PRJNA973832).

## Abbreviations

DAMs: Differentially abundant metabolites
DEGs: Differentially expressed genes
FPKM: Fragments per kilobase of transcript per million fragments mapped
AACT: Acetyl-CoA C-acetyl-transferase
HMGS: Acelyl-CoAC-acetyltransferase3-hydroxy-3-methylglutaryl-coenzyme A synthase
DXR: 1-deoxy-D-xylulose-5-phosphate reductor-isomerase
MCT: 2-C-methyl-D-erythritol-4-phosphate cytidylyl-transferase
HDR: 4-hydroxy-3-methylbut-2-enyl diphosphate reductase
MEP: 2-C- methyl-d-erythritol 4-phosphate
MVA: Mevalonate
CAS: Cycloartenol synthase
GAME: Glycoalkaloid metabolism
