# Supplementary material for: Metabolite profiling and transcriptomic analyses demonstrate the effects of biocontrol agents on alkaloid accumulation in Fritillaria thunbergii
Source: BMC Plant Biol. 2023 Sep 18;23:435. doi: 10.1186/s12870-023-04459-6 (PMC10506312; doi:10.1186/s12870-023-04459-6)
Supplement: Supplementary file 2 — Supplementary Material 2 [file 12870_2023_4459_MOESM2_ESM.docx]

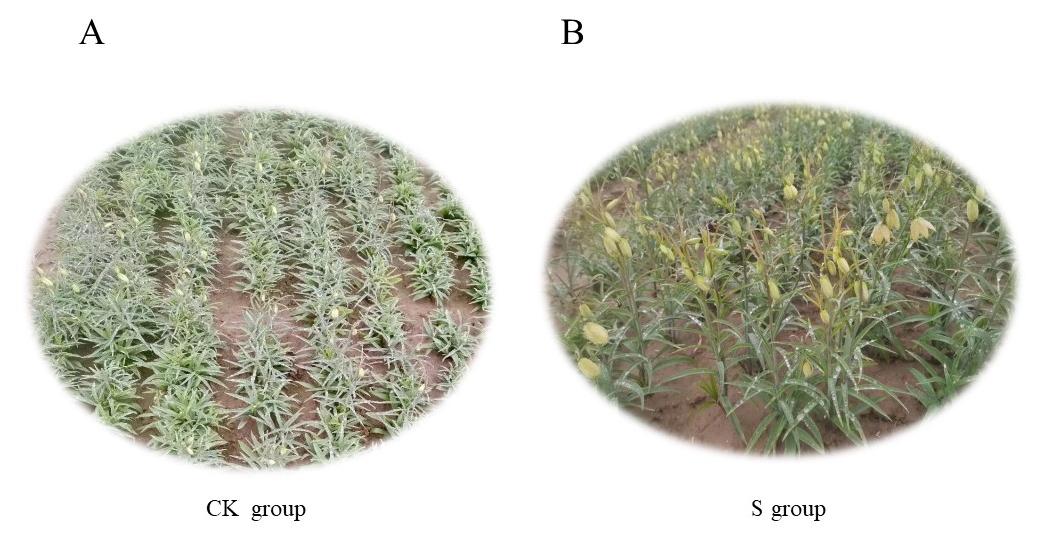
**Fig. S1.** The growth period of CK group(**A**) and S group(**B**) of *F. thunbergii*.


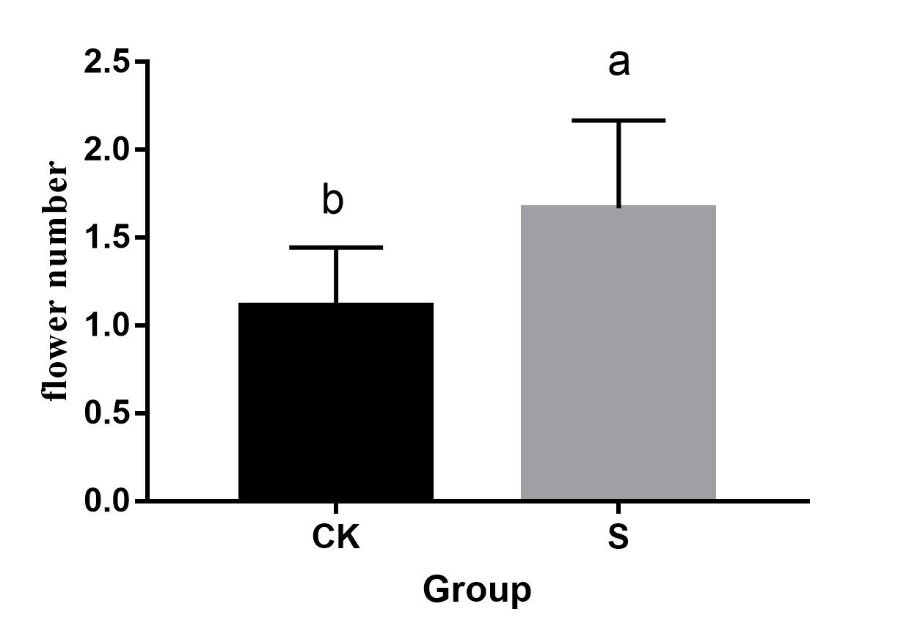


**Fig. S2.** The number of flowers per plant of *F. thunbergii* in growth period.


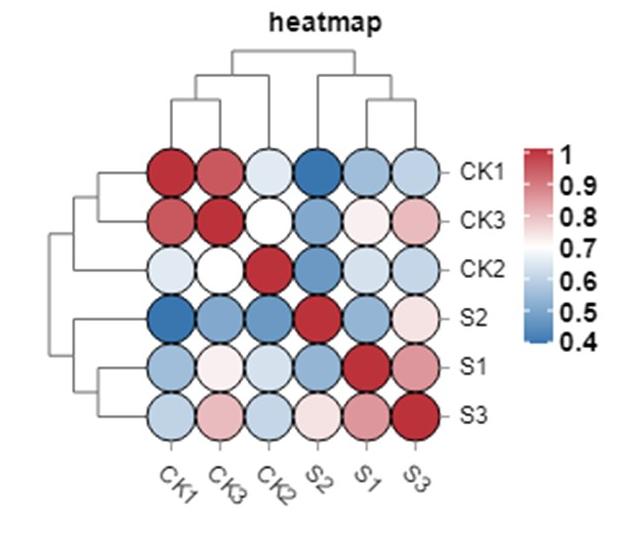


**Fig. S3.** Transcriptome-related heat maps of the CK and S groups.


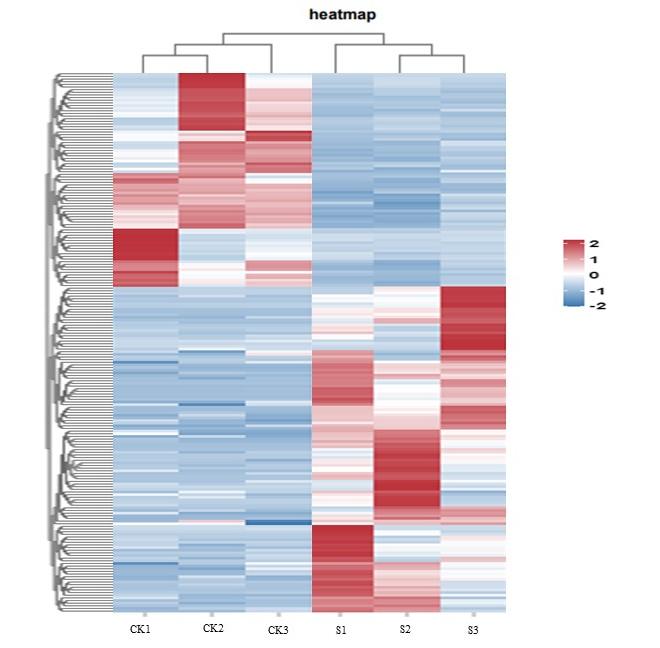


**Fig. S4.** Heat map of all the differential genes.
